# High Strain Rate Yielding of Additive Manufacturing Inconel 625 by Selective Laser Melting

**DOI:** 10.3390/ma14185408

**Published:** 2021-09-18

**Authors:** Kang Du, Laixia Yang, Chao Xu, Bin Wang, Yang Gao

**Affiliations:** 1 School of Mechanical Engineering, Xi’an University of Science and Technology, Xi’an 710054, China; 17101016003@stu.xust.edu.cn (K.D.); yanglx@xust.edu.cn (L.Y.); gaoyangyang@xust.edu.cn (Y.G.); 2 Department of Mechanical and Aerospace Engineering, Brunel University London, Uxbridge UB8 3PH, UK

**Keywords:** nickel-based superalloy, dynamics strength, strain rate, Hopkinson bar, selective laser melting

## Abstract

Nickel-based alloy Inconel 625, produced by the selective laser melting method, was studied experimentally for its mechanical performance under strain rate loading using Hopkinson bars. Both compression and tensile tests were carried out, with the former also being conducted at 500 °C. The strain rate was in the range of 300 to 3500 s^−1^ at ambient temperature, and 1200 to 3500 s^−1^ at the elevated temperature, respectively, for compression tests, and 900 to 2400 s^−1^ for tensile tests. Results show that the alloy has a strong rate sensitivity with the dynamic yield stress at 3500 s^−1^, almost doubling the quasistatic value. The test results also show that, even though the temperature elevation leads to material softening, the strain rate effect is still evidential with the dynamic compressive yield stress at the rate 10^3^ s^−1^ and 500 °C still being higher than the quasistatic one at ambient temperature. It is also observed that dynamic tensile strengths are generally higher than those of compressive ones at room temperature.

## 1. Introduction

Nickel-based superalloys are widely used in high-temperature applications, especially in the aerospace and energy industries. Due to its good structural stability, superior high-temperature strength, and oxidation resistance, it has become one of the irreplaceable components in high-temperature applications, such as turbine blades, engine discs, and gas turbines [1,2,3,4]. With the continuous development of aerospace technology, the function, structure and performance of its components have been put forward more stringent requirements, the integration of structure molding is conducive to improve the functional, lightweight, and mechanical properties of aviation components. For instance, nickel-based high temperature alloys are used extensively for engine blades. When they are hit by foreign objects, such as bird strikes or fractured pieces, high speed impact occurs and to the blades yielding in deformation under high strain rate loading. At present, most of the nickel-based alloy components are still produced by traditional casting and forging methods, which cannot easily produce integrated components with lightweight and complex geometries, restricting the development of aerospace technology [5]. The selective laser melting (SLM) method is one of the additive manufacturing technologies that has been developed rapidly in recent years. It has incomparable advantages over traditional manufacturing technologies, such as its integrated forming of complex structures, weight, topology optimization, and material utilization. The integrated additive manufacturing of producing customized materials, structures, and properties has significant engineering applications in aerospace and other fields [6].

SLM is one of the most rapidly developing additive manufacturing technologies in recent years. It has incomparable advantages in complex structure integration forming, weight, topology optimization, and material utilization. SLM creates an important way to integrate the manufacturing of power structures for the production of custom materials and complex components in fields such as aero engines and gas turbines [7]. Though SLM opens up a new approach to produce materials and components, as shown in the following Section on test specimen fabrications, the nature of laser melting passes involved in the process leads to inhomogeneity and anisotropy to certain extents, yielding varied material properties compared to those manufactured in traditional productions through casting, rolling or forging. The load bearing capacity and integrity of products fabricated by addictive manufacturing have been a concern by the research community and the industry [8,9].

Inconel is a registered trademark of Special Metals Corporation for a family of austenitic nickel-chromium-based superalloys [10], with Inconel 625 and 718 alloys being most commonly used by the industry. The mechanical property and the processing for SLM directly formed nickel-based alloys have also been studied extensively [11]. The results show that the static tensile mechanical properties of 3D printed Inconel alloys are close to those of forged parts under the condition of material density, but the dynamic failure research of 3D printing materials is still limited. Though there are some studies on Inconel 718, the performance of additive manufactured Inconel 625 under different strain rate loading, in particular, can hardly been seen in open literature.

Inconel 625 is a solid solution reinforced nickel-based superalloy widely used in aerospace and gas turbine fields. It is a nonmagnetic, corrosion and oxidation resistant nickel-based alloy. It has high strength and toughness in the temperature range up to 1100 °C, which is derived mainly from the solid solution effects of the refractory metals, niobium and molybdenum, in a nickel-chromium matrix [12]. 

The significance of this paper is to study the dynamic failure behavior of 3D printing Inconel 625 alloy. In order to evaluate the performance of SLM fabricated Inconel 625 under dynamic loading at ambient and high temperatures, both Hopkinson compressive and tensile bar systems were used. The effect of temperature on high strain rate behaviour is also considered by conducting tests at room temperature as well as at an elevated temperature. In addition, the effect of high strain rate on microstructure of the tested specimens is also observed [13,14].

## 2. Materials and Methods

### 2.1. Materials Characterization

**Figure 1:**
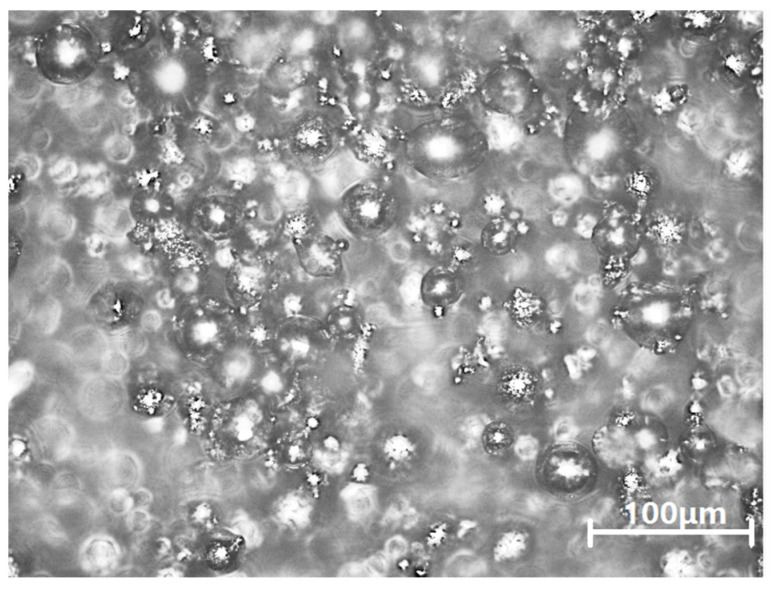
Inconel 625 Powder morphology.

Particle sizes of Inconel 625 powders (EOS Gmbh-Electro Optical System, Munich, Germany) used in this study range from 15 to 53 μm, with an average of 35 μm. Figure 1 shows the morphology of powder. Table 1 lists the chemical composition of the powders as provided by the manufacturer.

### 2.2. Specimen Design

Specimens for the compressive tests are in the shape of a thick circular disk, 4 mm in height and 8 mm in diameter, while the tensile ones are dumbbell shaped, with the gauge section of 5 mm in length and 5 mm in diameter. 

**Figure 2:**
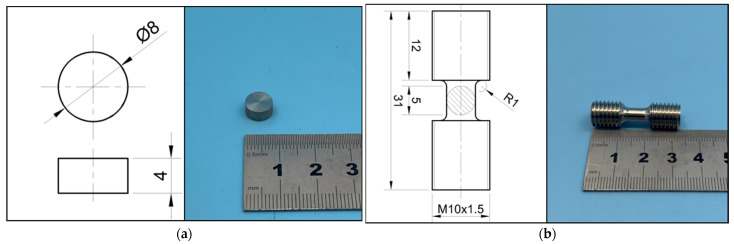
Dimensions of (**a**) compressive specimens and (**b**) tensile specimens. Unit: mm.

Dimensions of the specimen are shown in Figure 2. The surfaces of the compressive specimens were lightly grounded to the roughness of N4 for good contacts with the compressive bars. The one-dimensional stress wave theory of Hopkinson pressure bar determines that the action of pressure bar on specimen is plane loading, so the two ends of specimen are required to be parallel and smooth with the end faces of incident bar and transmission bar. The tensile specimens were threaded by machining for connection to the tensile bars.

### 2.3. Specimen Manufacture

**Figure 3:**
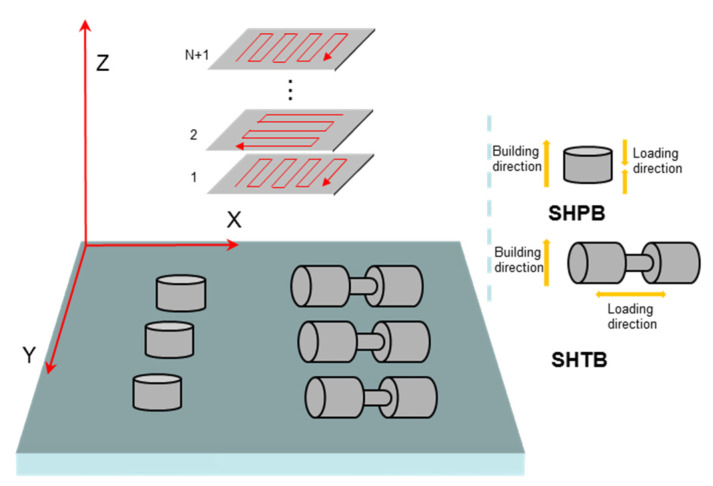
Printing directions and specimen layout.

The specimens were fabricated of BLT-S210, produced by BLT (Bright Laser Technologies Co., Xi’an, China). SLM process parameters used to produce the specimens are shown in Table 2. The specimens were printed to the net sharp directly in a powder bed, as shown in Figure 3. The laser scanning directions were in the horizontal X–Y plane, with build-up in the Z direction. The commonly used transverse raster scanning path was adopted to minimize inhomogeneity and anisotropy in microstructures and properties between printed layers. The preheating temperature before printing was 200 °C, the laser power was controlled at 380 W, the scanning speed was 500 mm/s, the scanning interval was 70 μm, the laser spot size was 60 μm, and the printing layer thickness was kept at 0.02 mm. The intended strain rate test directions are in Z for compression and X for tension [15,16].

All specimens fabricated were heat treated at 650 °C for 4 h to improve melting quality and reduce residual stresses. After the heat treatment, the average relative density of the specimens was measured using the immersion method using a glass measuring cut with water and a weight scale. The measured weight of the sample as *M*_0_, that of the glass with water as *M*_1_, and that of the sample in the watered glass as *M*_2_ were weighed, and the relative density of the sample *R* was calculated as

(1) $$\rho_{s}=\frac{M_{0}}{M_{2}-M_{1}}\;\times \;\rho_{H_{2}O}$$

(2) $$R=\frac{\rho_{s}}{\rho_{0}}\;\times \;100\%$$

$\rho_{s}$ is the actual sample density, $\rho_{H_{2}O}$ the water density, and *ρ*_0_ the theoretical density of the solid material. An average value of $\rho_{s}$ of 97.6% was taken after a minimum of 3 measurements.

### 2.4. Experimental Methods

#### 2.4.1. Quasi-Static Tensile Testing

**Figure 4:**
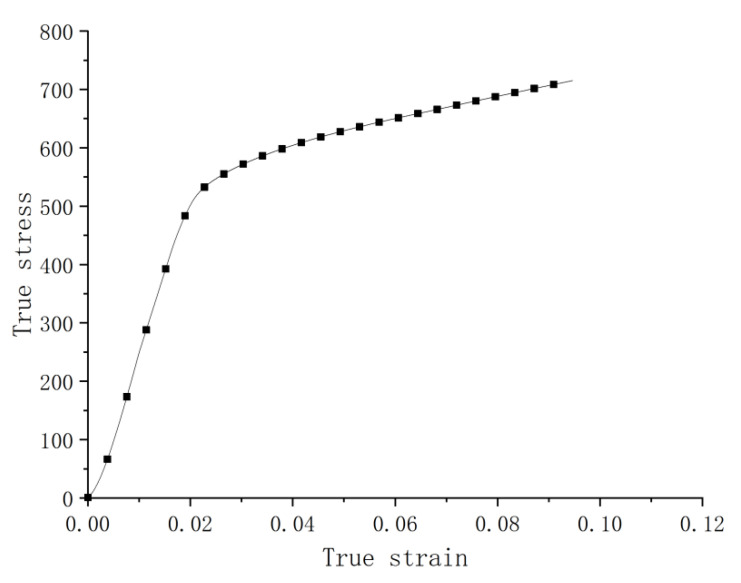
Stress–strain curve by tension at the strain rate 0.001 s^−1^.

A servo hydraulic universal testing machine was used for quasi-static tensile tests on the dumbbell shaped specimen. The loading rate was kept at 10^−3^ s^−1^. Figure 4 gives the stress–strain curve of the average of three tests. It illustrates an approximately bi-linear character over both the elastic and plastic ranges, apart from a slight J-shape at the beginning, probably caused by the small porosity. The 0.2% proof stress is seen as 540 MPa, and the corresponding yield strain is 0.022 (error band on the yield stress is shown in Section 4). 

#### 2.4.2. Split Hopkinson Compression Bar

**Figure 5:**
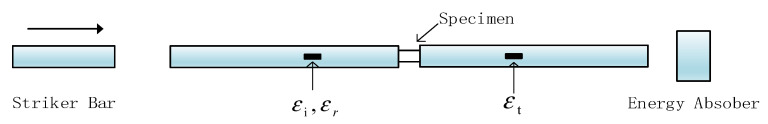
Layout of the Hopkinson compressive bar system.

The traditional Split Hopkinson pressure bar consists of an incident bar, a transmission one, and an impact one, as illustrated by Figure 5. The specimen is placed between the incident and the transmission bars. At the beginning of the test, the impact bar collides with the free end of the incident bar, and the stress wave generated by the impact propagates in the incident bar, reaching the specimen. Because of the impedance mismatch between the incident bar and the specimen, part of the wave is reflected, and the remaining stress wave passes through the specimen and deforms it, then is transmitted into the transmission bar as the projected wave. Strain gauges are used to measure the incident $\varepsilon_{i}$ and reflected waves $\varepsilon_{r}$ (both in the incident bar) and the projected wave $\varepsilon_{t}$ (in the transmission bar). Gauge readings are recorded by data logging through signal conditioning. The strain rate and stress data can then be calculated by Equations (3)–(5) using the classical one-dimensional stress wave theory [17,18]. Since relatively thin specimens are used in the Hopkinson bar experiment, stress propagation within the specimens is often ignored by assuming the stress balance in specimens.

(3) $$\dot{\varepsilon }=-2\frac{C}{L_{s}}\varepsilon_{r}$$

(4) $$\varepsilon =-2\frac{C}{L_{s}}\int_{0}^{t}\varepsilon_{r}\mathrm{dt}$$

(5) $$\sigma =\frac{A}{A_{s}}E\varepsilon_{t}$$

where $C=\sqrt{\frac{E}{\rho }}$, *L*_S_ is the initial length of the speciment, *ρ* the material density, and *E* the elastic modulus of the material. *A* and *A*_s_ are the cross-sectional areas of the bar and the specimen, respectively. In this study, the bars used were made from 18Ni steel with a diameter of 12.7 mm. Both the incident and transmission bars were 1220 mm long, and the impact bar was 400 mm long.

Compressive tests at the ambient temperature (25 °C) were carried out for strain rates at 300, 900, 1400, 2700, and 3500 s^−1^. Each rate was repeatedly tested twice. 

**Figure 6:**
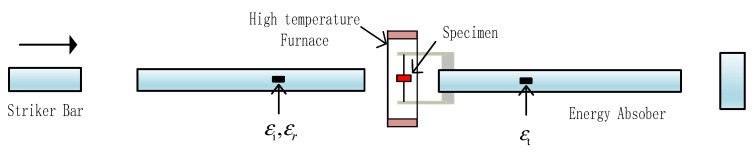
High temperature furnace used for the Hopkinson bar test.

Compressive tests at high temperature were also carried out. As the best long-term service temperature of nickel-based superalloy was up to 650 °C, with the maximum service temperature not exceeding 800 °C [19,20], the elevated temperature for this study was chosen as 500 °C. An electric resistance furnace was added to the compression test setup, as shown in Figure 6. The incident and transmission bars were first kept out of the furnace, separated from the heating process of the specimen, with the heating rate set at 5 °C/s. Additionally, after reaching the required temperature, it was held for 3 min. Then, both the incident and transmission bars were pushed to be in touch with the heated specimen; then, the test was carried out immediately. The strain rates achieved under the high temperature were 1200, 2000, 2600, and 3500 s^−1^, respectively.

#### 2.4.3. Split Hopkinson Tension Bar

**Figure 7:**
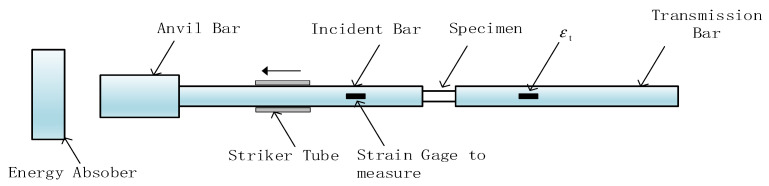
Set-up of Hopkinson tensile bar system.

The principle of the split Hopkinson tension bar (SHTB) system was the same as that of the compression one, but the connection of the bars, the specimen, and the pulse generation mode were different. As shown in Figure 7, both the incident and transmission bars were long tubes with internal threads at the corresponding ends to hold the dumbbell shaped specimen. The incident bar was rigidly connected to an anvil at the other end. When the striker tube outside the incident bar hit the anvil, a tensile wave was generated by the impact, which propagated in the incident bar towards the specimen. The remaining working principles were the same as those of the compressive bar system. The incident wave was first measured by the strain gauge. Once it reached the specimen, part of the wave was reflected back into the incident bar and measured again; the remaining part of the wave was transmitted through the specimen and deformed it, then into the transmission bar, which was also measured. The calculations were also performed using Equations (1)–(3) [21]. In this study, rates of 900, 1400, 1800, and 2400 s^−1^, respectively, were achieved in the tensile tests at ambient temperature. 

### 2.5. Microscopy of Original Specimens

**Figure 8:**
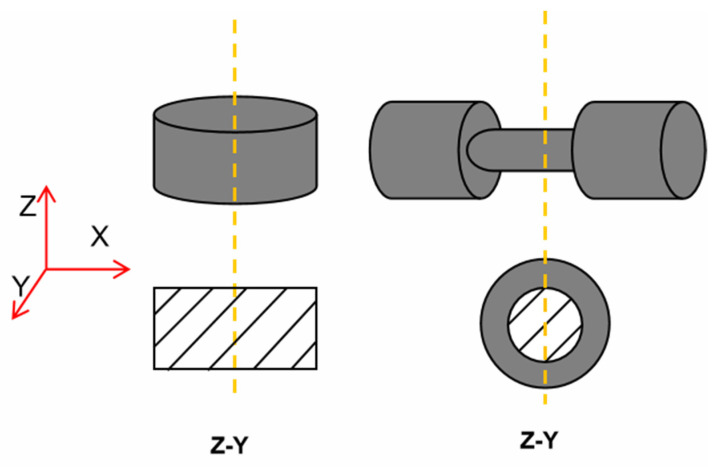
Section cutting diagram.

The metallographic structure was observed with a Smartproof5 optical microscope. The specimen was cut along the axial center of the compressed specimen by the method of wire cutting, as shown in Figure 8. After grinding and mechanical polishing of the profile, a corrosive agent with a ratio of 10 g FeCl_3_ + 90 mL HCl was used to corrode the specimen after grinding for 10 s. Because the nickel-based alloy has strong corrosion resistance, a 20% H_2_SO_4_ + 80% CH_3_OH (volume fraction) electrolyte was used for electrolytic polishing. The DC voltage was 20V, and the polishing time was 30 s. 

**Figure 9:**
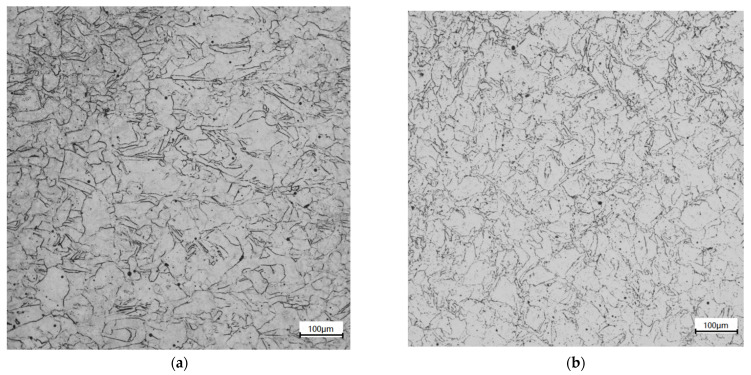
Metallographic structure of original time section. Experimental results (**a**) compression specimen, (**b**) tensile specimen.

As shown in Figure 9, the grains in SLM alloy are distributed in a checkerboard pattern, with most of them being of irregular shapes. Larger grains exist, and smaller grains are distributed at the edge of larger grains. These grains are elongated and twinned.

## 3. Experimental Results

### 3.1. Dynamic Tests

#### 3.1.1. Split Hopkinson Compression Bar Result

**Figure 10:**
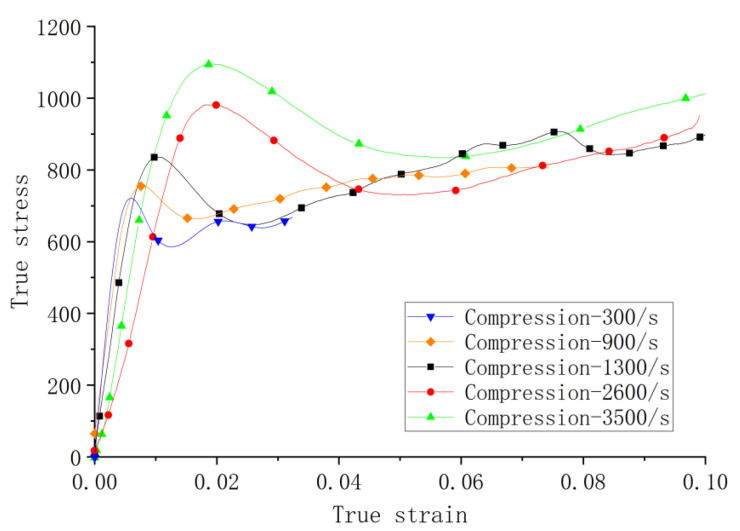
Stress–strain curves of compression tests at ambient temperature.

**Figure 11:**
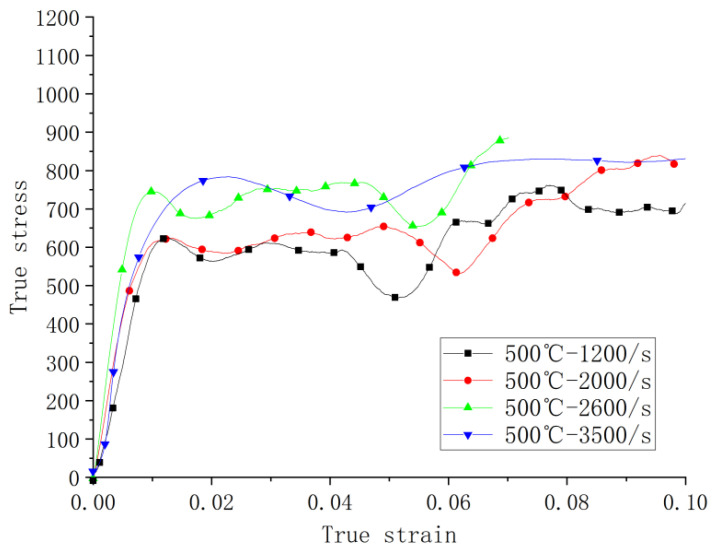
Stress–strain curves of compression tests at 500 °C.

**Figure 12:**
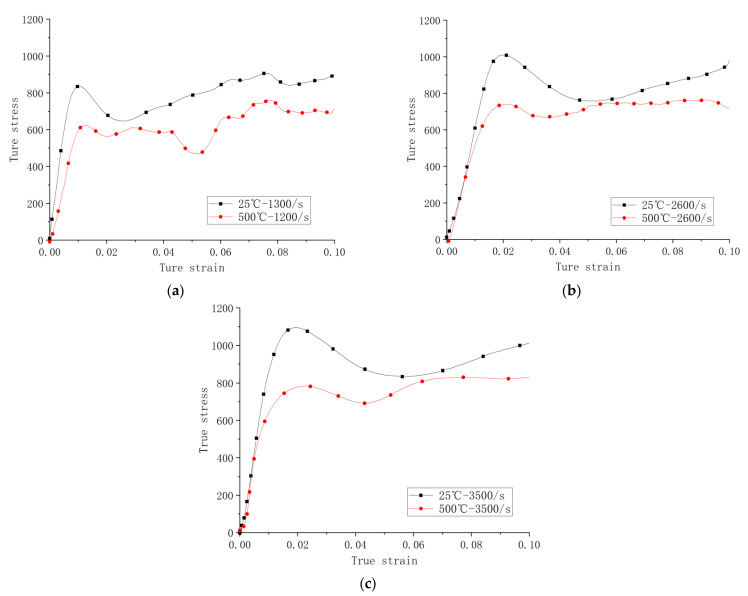
Stress–strain curves of dynamic compressive tests (**a**) at ambient temperature under 1300 s^−1^ and at 500 °C under 1200 s^−1^, (**b**) at ambient temperature and 500 °C under 2600 s^−1^, and (**c**) at ambient temperature and 500 °C under 3500 s^−1^.

**Figure 13:**
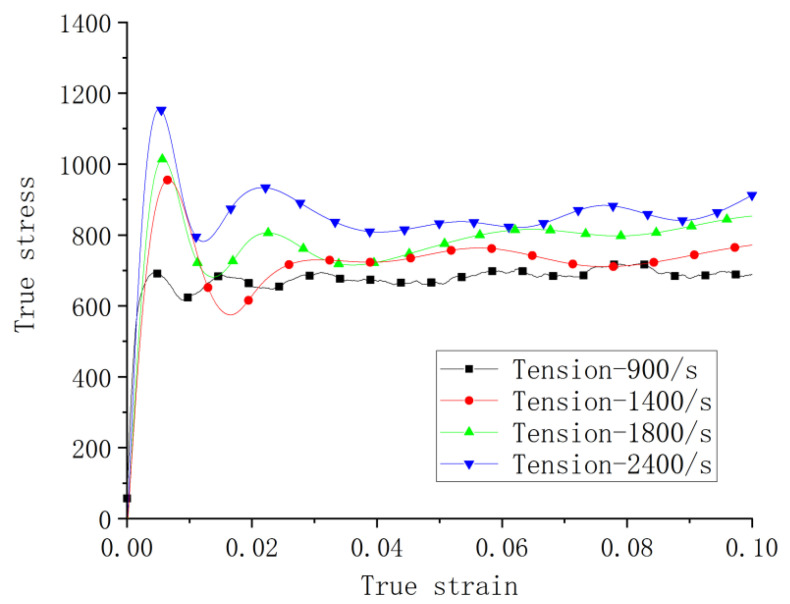
Stress–strain curves of different strain rates in dynamic tensile tests at room temperature.

**Figure 14:**
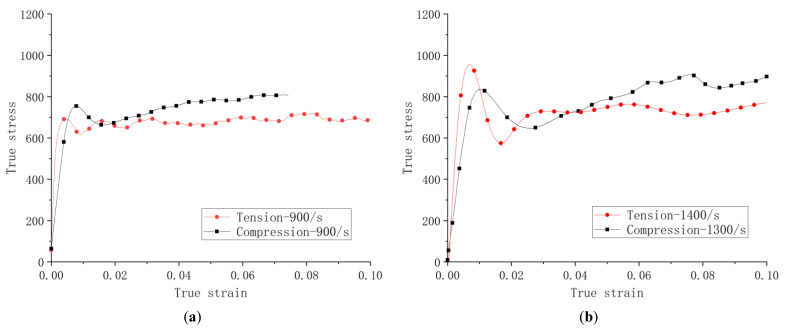

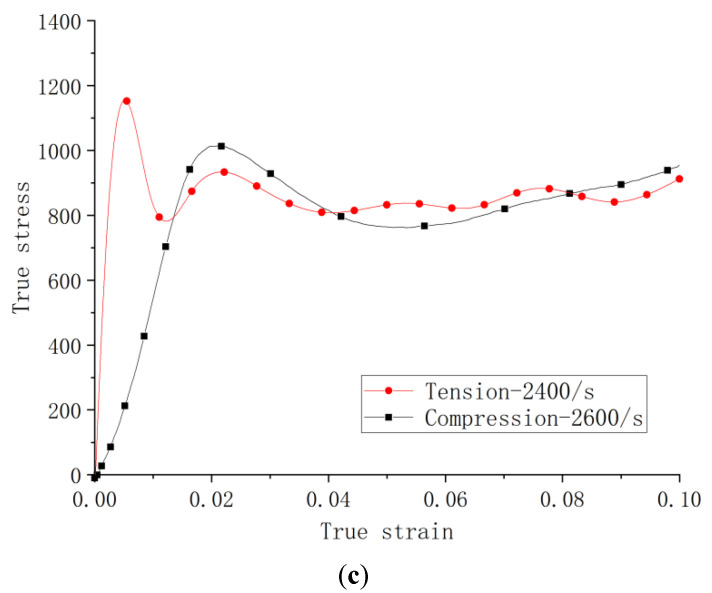
Stress–strain curves of (**a**) compression and tension at 900 s^−1^, (**b**) compression at 1300 s^−1^ and tension at 1400 s^−1^, and (**c**) compression at 2600 s^−1^ and tension at 2400 s^−1^.

The experimental results are shown in Figure 10, Figure 11, Figure 12, Figure 13 and Figure 14. Figure 10 illustrates the true stress–strain curves of compression tests under various strain rates at the ambient temperature. It can be seen that the dynamic stress–strain curve of Inconel 625 starts with a linear elastic response and is followed by a nonlinear increase to the peak stress; then, it softens and regains strain hardening. Collectively, curves at different strain rates tested show an increasing strength in terms of the strain rate, indicating a clear rate sensitivity by the material. The increase in the first peak stress is consistent, but the slope of the elastic response is not, indicating some variation in Young’s modulus. The variation could be due to the relative density ratio of the specimens being at 97.6%, with voids and scattering microstructures due to the nature of addictive manufacturing process. Further investigation is clearly needed in this aspect.

In the final hardening stage, test curves also show some wavy phenomenon, which may be related to dynamic recrystallization due to the energy stored inside the specimen reaching a critical value after deformation. Rolling average and wave filtering techniques were used to reduce curve oscillations. Note that only one test curve is shown, but all tests were repeated at least twice to estimate the error bands for the yield stress (see Section 4).

Figure 11 shows the results of the dynamic compression tests at 500 °C. Stress–strain curves show similarity to those under the ambient temperature with increasing strengths in terms of strain rate. The material still shows rate sensitivity with the increase in strain rate at this temperature. Additionally, there are differences in the slope of the elastic stage, indicting data scattering and some variations in the Young’s modulus. 

The overall strengths at the high temperature are lower compared with those at the ambient temperature for the strain rates of the same or close values. This can be more clearly seen in Figure 12 where the corresponding curves at the ambient and high temperatures are paired at the closest strain rates. The thermal softening effect on yielding is evidential for all the strain rates tested. The thermal effect includes coarser grains and the secondary metallurgical reaction during the process of receiving compression, resulting in the decrease in the strength.

All compression specimens were recovered after the test and observed for deformation. No barrowing effect was identified in all specimens. The specimen surfaces were found to become rougher, particularly in those tested under high temperature, with some of them showing micro cracks. A detailed study on the evolution of microstructures as the effect of the high strain rate loading under both ambient and high temperatures are being conducted and will be reported separately [22,23].

#### 3.1.2. Split Hopkinson Tension Bar Results

Dynamic tensile tests were all carried out at the ambient temperature. Figure 13 shows the stress strain curves at 900, 1400, 1800, and 2400 s^−1^, respectively. It can be seen that the peak stress significantly to the strain rate, showing rate sensitivity under tension. The following extended plastic stage shows a more plateaued nature in comparison to the hardening character in curves of the dynamic compression tests at the ambient temperature. All specimens were examined after the dynamic tensile tests, and no necking was observed.

Figure 14 shows the comparisons of the corresponding dynamic compression and tensile test curves at ambient temperature at the closest strain rates. The slope of the elastic range of the dynamic tensile curves are higher than that of the compressive ones. Additionally, the dynamic tensile curves also seem to show less hardening in the extended plastic.

### 3.2. Microscopy Observation of Specimens under Dynamic Compression

**Figure 15:**
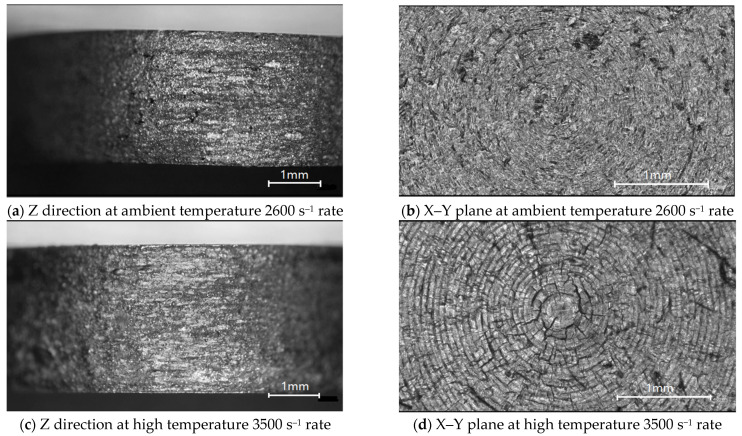

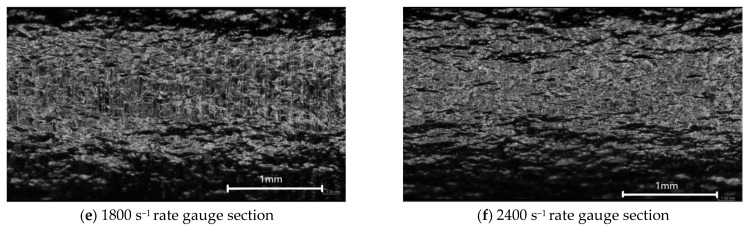
Microscopy observations of tested specimens.

Magnified images of the surfaces of the specimens under two compressive strain rates are shown in Figure 15. In Figure 15a,c, at strain rates of 2600 and 3500 s^−1^, the compression in the Z direction is obvious, and the corresponding reductions in length are 20.7 and 30.5%. Diameter increases are 14.2 and 20.2%, respectively. 

With the increase in the strain rate, poorer ductility was observed. This is shown in Figure 15b,d, where few fractures are observed in the former, but multiple cracks are clearly visible in the latter.

Figure 15e,f show the gauge section of tensile specimens under 1800 and 2400 s^–1^ strains. The original gauge section is 5 mm long and 5 mm in diameter. After stretching, the gauge section increases are 9.2 and 13%, and the diameter is shortened by 10.7 and 15.7%. The tensile specimens showed no necking phenomenon and obvious crack [24,25].

**Figure 16:**
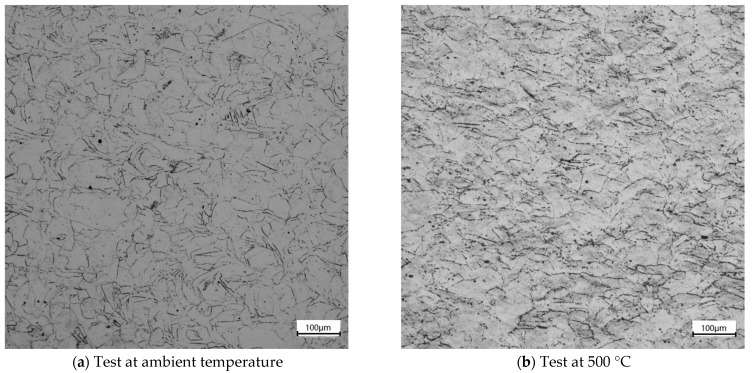
Metallographic structure of tested specimens of compression at 3500 s^−1^.

The changes in metallographic microstructure for the test of 3500 s^−1^ at the ambient and high temperatures were observed, as shown in Figure 16. The austenite grain structure is seen as irregular and elongated, and there are obvious twinned grain structures in the austenite grain, with a small number of voids in the specimen. It shows that the laser additive manufacturing technology has the characteristics of fast cooling speed, which traps gas in the molten pool and eventually leads to the formation of void defects after solidification. There is a small amount of precipitate at the grain boundary as well [26]. 

**Figure 17:**
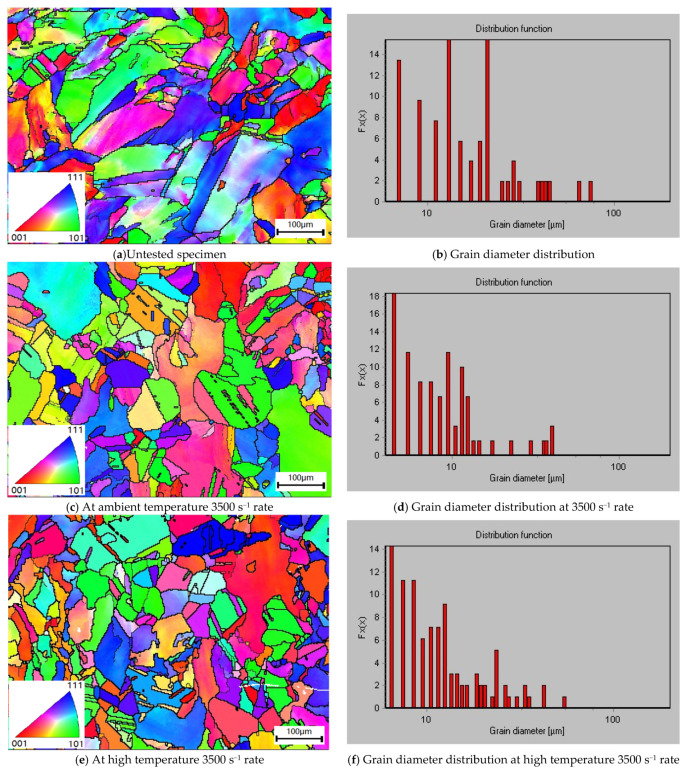
EBSD microscopy of dynamically compressed specimens.

EBSD microscopy was used to measure the grain size of the specimen. Figure 17 shows the morphology and grain size distribution of the untested specimen and those compressed dynamically at 3500 s^−1^ under the ambient and high temperature, respectively. The average grain sizes are approximately 17.53, 14.91, and 10.84 μm, respectively, showing an average grain size reduction of 15 and 28.2%, respectively, in the two tested specimens. Because the laser-melted Inconel 625 alloy belongs to the lower-level fault-energy material, and dislocation is easy to decompose into lamination, the width of extended dislocation is large, difficult to bundle and slip, and prone to twinning deformation. The grain refinement of the alloy is mainly affected by the dislocation network and deformation twin division mechanism, yielding in decreasing grain size in terms of the strain rate and compression deformation.

## 4. Discussion on the Dynamic Yield Stress

**Figure 18:**
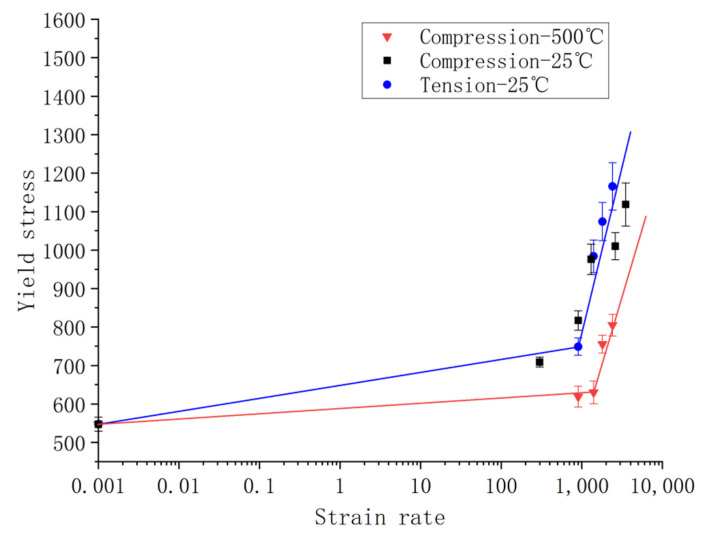
Dynamic yield stress vs. strain rate.

Figure 18 is a schematic diagram of the dynamic yield strengths of Inconel 625 in semi-logarithmic scale at different strain rates from the compression and tensile tests at the ambient temperature, and from the compression tests at 500 °C, respectively. Error bars were obtained based on repeated tests. 

It can be seen from the figure that the dynamic yield strength at the ambient temperature only increases moderately for the strain rate up to 10^3^ s^−1^. For instance, at 300 s^−1^, the dynamic yield stress under compression is only 30% higher than that of the quasistatic one. However, the dynamic yield stress increases rapidly when the strain rate is above the range of 10^3^ s^−1^, with its value being more than double that of the quasistatic one when the strain rate is 2.6 × 10^3^ s^−1^.

Similarly, high temperature tests at 500 °C show only slight increases in the dynamic yield stress up to 10^3^ s^−1^; then, a more evidential increase in the yield stress is shown, nearly 50% higher than the quasistatic one. The effect of strain rate sensitivity is “weakened” by the elevated temperature in comparison to that in the ambient temperature; nevertheless, it is still significant.

On the whole, it can be seen that the dynamic yield strength of Inconel 625 increases rapidly with the strain rate, regardless of being loaded in compression or tension. Inconel 625 shows a clear sensitivity to the strain rate. There is a bi-linear character in the trend of change in the dynamic yield stress as a function of the strain rate, as commonly seen in metals sensitive to the strain rate. The kink point of the linear “curve-fitting” is seen to be approximately 10^3^ s^−1^. Once the strain rate is beyond this value, a higher slope of the rate sensitivity is shown. Note that, as the test points of the dynamic compression and tensile loading at the ambient temperature are close, the material can be approximately treated as “isotropic”, and one single bi-linear fitting is there given for ambient temperature tests [27,28].

## 5. Conclusions

This experimental study was carried out on 3D printing Inconel 625 specimens with both dynamic compression and tensile tests at the ambient temperature and dynamic compression tests at 500 °C. The following conclusions can be drawn from the test results:

1. The dynamic yield stress of Inconel 625 is influenced markedly by the strain rate loading with its value becoming significantly higher at high strain rates than that under quasi-static loading.
2. At the ambient temperature, the dynamic yield strength of the material under tension appears to be slightly higher than that under compression test at the same or similar strain rate.
3. At the same or similar strain rate, a high temperature yields in a lower dynamic yield stress compared to that at the ambient temperature.
4. The relationship between the dynamic yield stress of Inconel 625 and the strain rate can be approximately described as bi-linear, for which a “curve fitting” exercise can be taken to quantify the relationship if needed.
5. The post yield dynamic behaviour of Inconel 625 poses to be strain hardening under dynamic compression, but attenuation to a plateau under dynamic tension.

## Figures and Tables

**Table 1:** Chemical composition (wt.%) of Inconel 625 powders.

Ni	Cr	Mo	Fe	Nb (Cb)	Nb (Cb) + Ta	Co	W	Mn	Si	Cu	Al	Ti
≥58	20–23	8–10	≤5.0	3.15–4.15	3.15–4.15	≤1.0	0.1–1.0	≤0.05	≤0.5	≤0.05	≤0.4	≤0.4

**Table 2:** SLM process parameters used for specimen fabrication.

Laser Power (w)	Scanning Velocity (mm/s)	Scanning Interval (μm)	Spot Diameter (μm)	Increment in Z (μm)
380	500	70	60	20

## Data Availability

The data presented in this study are available on request from the corresponding author.
